# A multidisciplinary perspective on the complex interactions between sleep, circadian, and metabolic disruption in cancer patients

**DOI:** 10.1007/s10555-021-10010-6

**Published:** 2021-12-27

**Authors:** Lasse D. Jensen, Delmy Oliva, Bengt-Åke Andersson, Freddi Lewin

**Affiliations:** 1 Department of Health, Medical and Caring Sciences, Unit of Cardiovascular Medicine, Division of Diagnostics and Specialist Medicine, Linköping University, Campus US, Entrance 68, 58185 Linköping, Sweden; 2 Department of Oncology, Ryhov County Hospital, 55185 Jönköping, Sweden; 3 Department of Clinical and Experimental Medicine, Linköping University, 58185 Linköping, Sweden; 4 Department of Laboratory Medicine, Region Jönköping County, 55185 Jönköping, Sweden

**Keywords:** Sleep, Cancer, Circadian clock, Metabolism, Treatment, Patient care, Treatment

## Abstract

Sleep is a basic need that is frequently set aside in modern societies. This leads to profound but complex physiological maladaptations in the body commonly referred to as circadian disruption, which recently has been characterized as a carcinogenic factor and reason for poor treatment outcomes, shortened survival, and reduced quality of life in cancer patients. As sleep and circadian physiology in cancer patients spans several disciplines including nursing science, neurology, oncology, molecular biology and medical technology, there is a lack of comprehensive and integrated approaches to deal with this serious and growing issue and at best a fractionated understanding of only part of the problem among researchers within each of these segments. Here, we take a multidisciplinary approach to comprehensively review the diagnosis and impact of sleep and circadian disruption in cancer patients. We discuss recent discoveries on molecular regulation of the circadian clock in healthy and malignant cells, the neurological and endocrine pathways controlling sleep and circadian rhythmicity, and their inputs to and outputs from the organism. The benefits and drawbacks of the various technologies, devices, and instruments used to assess sleep and circadian function, as well as the known consequences of sleep disruption and how sleep can be corrected in cancer patients, will be analyzed. We will throughout the review highlight the extensive crosstalk between sleep, circadian rhythms, and metabolic pathways involved in malignancy and identify current knowledge gaps and barriers for addressing the issue of sleep and circadian disruption in cancer patients. By addressing these issues, we hope to provide a foundation for further research as well as better and more effective care for the patients in the future.

## Introduction

**Fig. 1:**
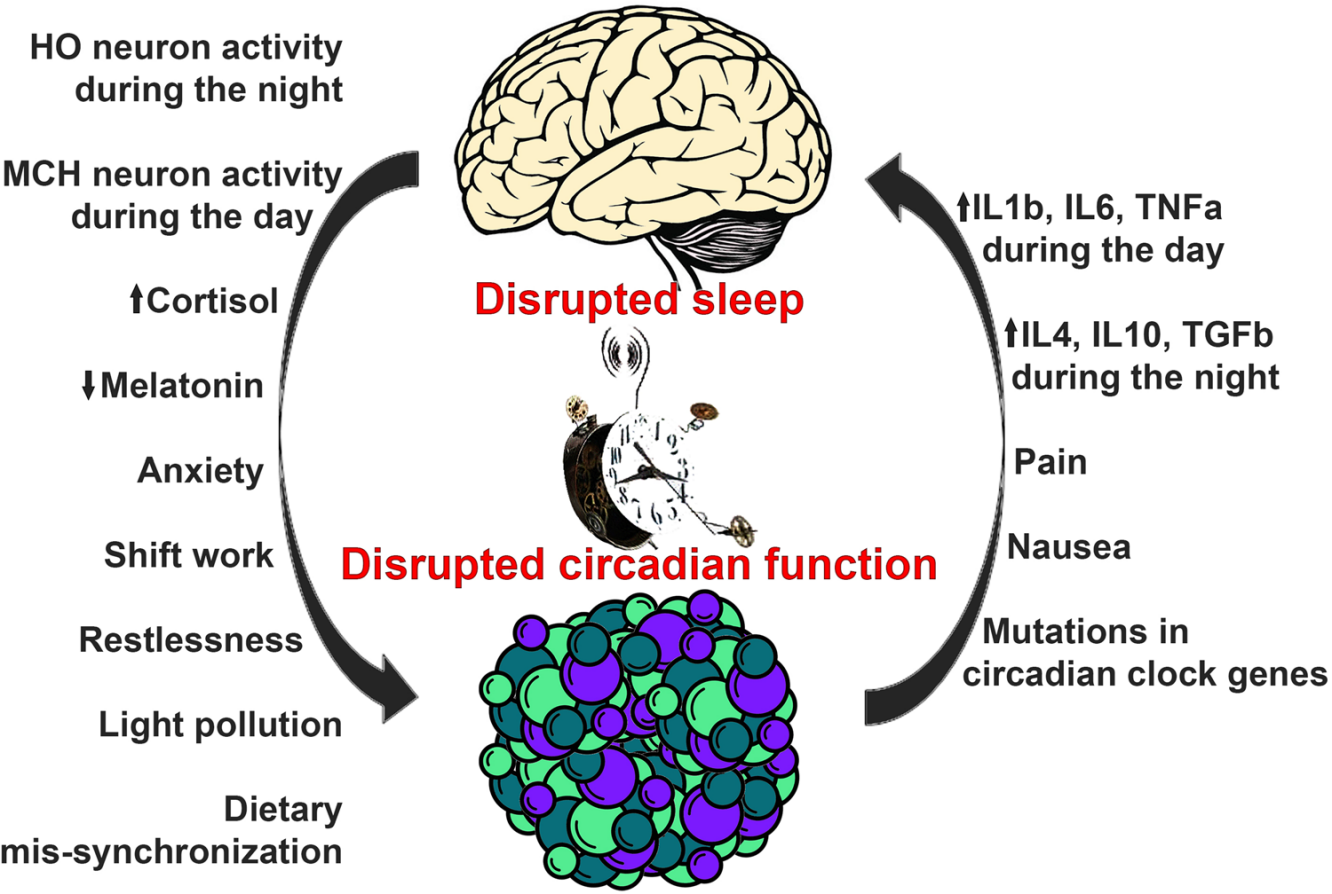
Vicious circle underlying sleep disruption in cancer patients. Poor sleep due to shift work, jet lag, anxiety, restlessness, and light pollution is associated with increased HO-neuron activity during the night and increased MCH-neuron activity during the day. This leads to increased cortisol and reduced melatonin secretion, as well as dietary mis-synchronization, which increase the risks of cancer, tumor growth, metastasis, and treatment failure. Cancer-associated mutations in clock proteins and low-grade inflammation, as well as pain and nausea which are also common side effect of chemotherapy, lead to deregulated cytokine production. Increased IL1β, IL6, and TNFα during the day are associated with daytime sleepiness, and increased IL4, IL10, and TGFβ during the day are associated with insomnia. These inflammatory factors also lead to BBB-disruption and disrupted regulation of sleep centers in the brain

Insufficient sleep quality and/or duration has become one of the most prevalent, elective risk factors for cancer as well as treatment failure and poor quality of life in cancer patients[1]. While the reasons for these effects of poor or little sleep are poorly understood, it is established that insufficient sleep and/or irregular sleep patterns such as seen in people frequently working shifts affect cancer initiation, progression, and treatment [2]. This occurs mainly through disrupting physiological circadian rhythms, biological processes that are normally engaged in a rhythmic manner, and with a frequency of approximately 24 h. Sleep disruption affects circadian function at all biological scales including interfering with distinct molecular interactions inside the cell, which affects cellular physiology, tissue homeostasis, and the physiology of the whole organism [1]. Circadian dysfunction due to sleep disruption can commonly be classified by five main criteria (Fig. 1): (1) behavioral sleep disruption also known as social jet lag, occurring due to social or occupational shifts in the active period from the day to the night or vice versa, or poor night-time sleep quality due to for example alcohol or substance (ab)use [3]; (2) frequent jet lag which arise from traveling across multiple time zones [4]; (3) anxiety, noise or light pollution, or insomnia due to other reasons related to restlessness of the body or mind [2]; (4) pain, nausea, or other types of non-psychological discomfort [2]; and (5) genetic disruption of genes involved in circadian rhythmicity or sleep [5]. In all these cases, sleep disruption, through dysregulation of the circadian clock, leads to an inability of the organism to maintain the circadian rhythm of key biological functions including metabolism, angiogenesis, immunity, DNA repair, and cell cycle regulation, all being hallmarks of cancer [1, 2, 6]. Indeed, shift work was in the Nurse’s Health Study, a large epidemiological study including almost 200 000 individuals and 24 years of follow-up, found to increase the risk of breast cancer by 2.2 fold, particularly when shift work started as a young adult [7], and was in 2019 recognized by the International Agency for Research on Cancer as probably carcinogenic to humans (Class 2A) [8]. While sleep disruption leading to circadian dysfunction is also a common and growing problem in the general population, with an aggregated prevalence of approximately 20% [3], this is likely one of the most important risk factors for cancer today.

While shift work is an important and highly studied mechanism for circadian disruption leading to increased cancer risk in humans, other factors such as light pollution during the night or chronic jet lag due to other reasons may also play important roles. To address the issue of light pollution, studies have investigated the risk of cancer among people who live in urban areas characterized by intense night-time illumination or people who sleep with the indoor lights on (reviewed by Walker et al. [9]). While the results from these studies are not consistent, the overall trend implies that light pollution poses an increased cancer risk, which has also been validated in large meta-analyses and reviews of the available data [9]. In animal models, shift work is often studies by exposing the mice to shortened dark periods every other night, leading to a condition known as chronic jet lag. Such treatments are associated with an increased probability and speed of breast cancer development, perhaps most clearly demonstrated in the MMTV:PyMT mouse model [10]. These preclinical findings lend further evidence to the general carcinogenic effect of chronic circadian disruption such as that associated with shift work in humans.

### Prevalence and consequences of sleep disruption in cancer patients

In addition to increasing the risk factor for cancer, sleep and circadian disruption is also found, and at an increased prevalence and severity, after disease onset. According to recent meta-analyses, the prevalence of sleep disruption is at least three times higher [11, 12] in cancer patients compared to the general population, with breast cancer patients being at particularly high risk [12]. Due to a lack of common, consensus guidelines and diagnostic criteria, the specific prevalence of sleep disruption in cancer patients, however, is highly study-specific and depends on among other things the instrument and cut-off levels used for diagnosis. Palesh et al. [12], using the Hamilton Depression Inventory, reported prevalence rates higher than 80% for breast cancer patients experiencing “some insomnia symptoms,” although only half of these met clinical criteria for insomnia. On the other hand, Phillips et al. [13], using a modified version of the Pittsburgh sleep quality index, found the prevalence to be as low as 26% (i.e., close to the general population level) for an aggregated population of cancer patients. Importantly, however, sleep disruption may extend far into survivorship at similar prevalence as in the patient population (up to 65%) [14].

Sleep disruption in cancer patients is closely associated with poor prognosis and treatment failure. Evidence supporting large differences in survival among patients with healthy sleep habits compared to patients exhibiting lack of sleep or poor sleep quality has been accumulating during the last decade. Innominato et al. demonstrated in a landmark study that patients receiving chemotherapy for metastatic colorectal cancer and being at rest during their time in bed, a common method to objectively evaluate sleep, gained 8 additional months of overall survival (OS) compared to those that were frequently moving during their time in bed (OS: 22.3 months compared to 14.7 months) [15]. This survival benefit is significantly larger than the approximately 3 months achieved by medical treatment in most clinical trials done in this patient group [16], clearly demonstrating the importance of ensuring good sleep hygiene of the patients. Furthermore, medical treatments should be provided at a time that ensures peak plasma concentrations coincide with the peak activity of the targeted process—for example, DNA-modulating therapies should be given in the evening to ensure peak plasma concentrations during the night when DNA synthesis and repair pathways are active in the cancer cells [17]. These findings have led to the concept of chronomodulated therapy or chronotherapy, which is now gaining increased attention within medical oncology. In addition to improved efficacy, chronomodulation may also consider drug metabolism and the timing of drug-targeted processes in healthy cells [17], such that drugs can be administered at a time when side effects are minimal. As the side effects are often severe and may lead to sleep disruption on their own [18, 19], giving drugs at a time when they a best tolerated should become a primary consideration when evaluating chronomodulation of therapeutic interventions in the future.

### The cellular clock

**Fig. 2:**
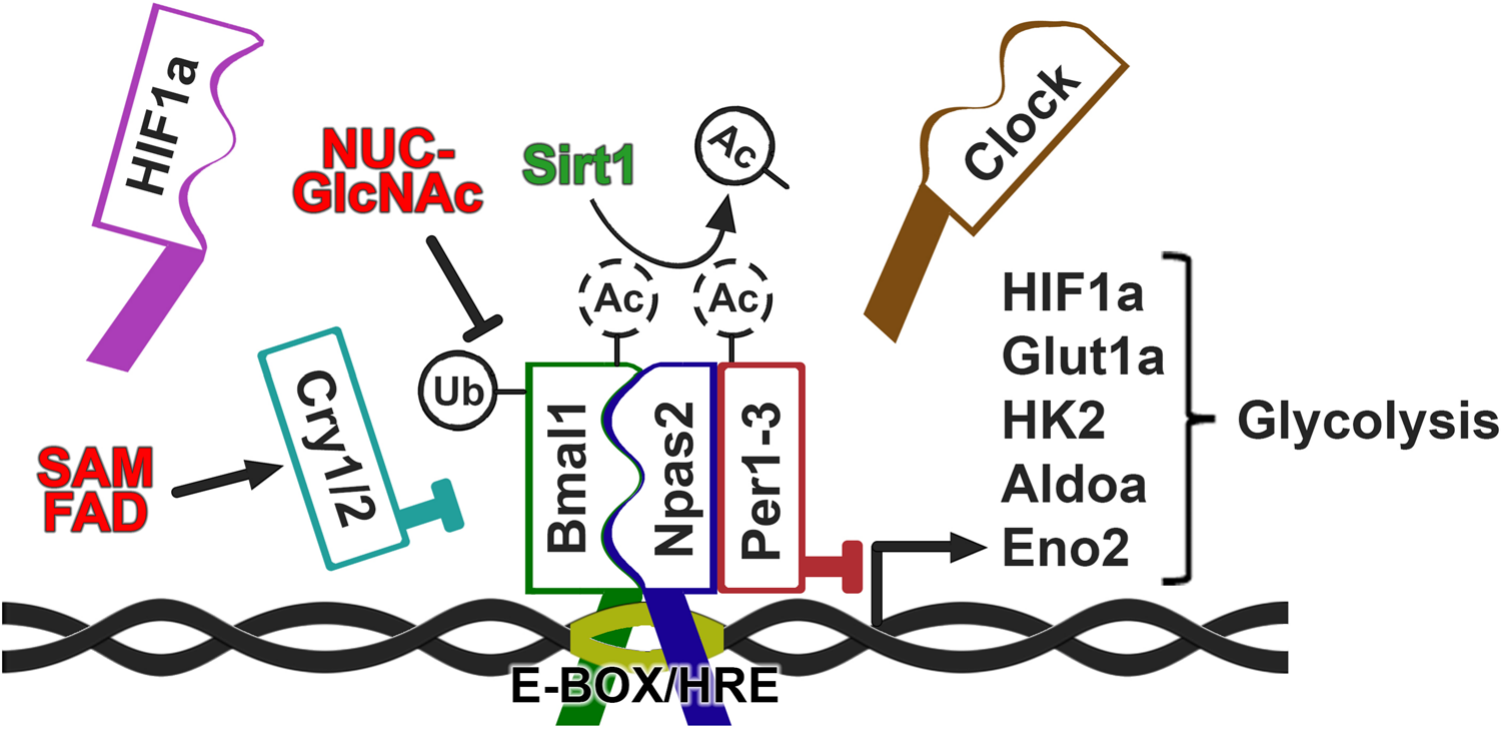
Circadian and metabolic crosstalk in cancer cells. The core clock factors Bmal1, Npas2, Clock, Hif1a, Per1-3, and Cry1/2 are required at oscillating concentrations during the day and night to maintain circadian homeostasis. Activators (Bmal1, Npas2, Clock, Hif1a) bind E-boxes/hypoxia-responsive elements (HRE) sequences in the promoter region to induce glycolysis during the day. Repressors (Per1-3 and Cry1/2) inhibit this during the night, allowing oxidative metabolism. Expansion of daytime metabolism due to deletion of Per, or Sirt1 (proteins important for stabilization of clock rhythmicity), increased glycolytic flux leading to elevated NUC-Glc-NAc, increased levels of FAD and SAM, or stabilization of HIF1a, results in persistent expression of glycolytic enzymes (Glut1, HK2, Aldoa, Eno2) and thereby maintained glycolysis during the night

Rhythmic oscillations in physiology and behavior controlled by the suprachiasmatic nucleus in the hypothalamus and peripheral circadian clocks leads to circadian changes in pharmacokinetics and pharmacodynamics of anticancer drugs [20]. Such chronopharmacokinetics and chronopharmacodynamics, collectively referred to as chronopharmacology, form the basis for chronomodulation of therapy [21]. The peripheral clocks involved in chronopharmacology are constructed at the molecular level from a relatively simple negative transcription-translation feedback loop in which the transcriptional activators Bmal1, Npas2, and Clock are inhibited by the transcriptional repressors of the Period and Cryptochrome families, which are induced by Bmal1/Npas2/Clock (Fig. 2). Bmal1, Npas2, and Clock, which constitute the motor of the cellular clock, are all belonging to the basic helix-loop-helix (bHLH) family that share high similarity to the hypoxia-inducible factors (HIFs) [22–24]. Indeed, Bmal1, Npas2, and Clock form heterodimers that bind DNA-elements known as E-boxes (consensus sequence: CACGTG), containing within them the hypoxia-responsive element (consensus sequence: ACGTG) bound by HIF-1 or -2 transcription factors [23]. Circadian clock factors including Bmal1 and Clock may also bind HIF1α and –β [25, 26], further adding to the extensive crosstalk and cross-regulation between the circadian clock and the hypoxia-responsive system. This crosstalk is contributing to the role of the circadian clock in the regulation of cellular metabolism. Bmal1 is an essential heterodimerization partner as Npas2 and Clock cannot form a transcription activation complex on their own [27]. Consequently Bmal1-knockout mice are devoid of cellular circadian regulation [27]. Bmal1 and one of its heterodimerization partners bind to E-boxes in the promoters of all clock-controlled genes (CCGs), which are approximately 50% of all protein coding genes [1], and activate their transcription. Among these are genes of the Period and Cryptochrome families, the protein products of which bind to the Bmal1-Clock or Bmal1-Npas2 complex and act as transcriptional repressors. After a period of Bmal1-Clock/Npas2 activity, Per and Cry will therefore accumulate and exert feedback-inhibition essentially shutting down further clock-controlled gene transcription. Cry1 and Per2 also bind to and respectively repress [28] or augment [29] the transcriptional activity of HIF1α, leading to an additional control by the circadian clock over hypoxia-associated cellular metabolism. As all of these transcription factors and repressors are rapidly degraded by ubiquitin ligases, but because Bmal1 is regulated by a different, auxiliary pathway activated by RORα, Per, and Cry levels will soon diminish, while Bmal1 levels will rise and eventually reinitiate the cycle [30]. The duration of this cycle requires various other factors that either post-transcriptionally modify these core clock proteins affecting their ability to bind heterodimerization partners, are involved in building the transcriptional complex, or transiently bind them in the cytosol to restrict their translocation to the nucleus [22, 30]. The transcription of the gene encoding the essential activator Bmal1 is activated by RORα through external signals such as dietary metabolites and cortisol during the active period or melatonin produced by the pineal gland during sleep in the dark [31] (Fig. 1). Most of the dietary metabolites are produced rhythmically by the liver, and the melatonin production is under the control of the suprachiasmatic nucleus (SCN) in the brain, receiving light signals through the retino-hypothalamic tract. As such, the liver and SCN are considered the two main circadian “pacemakers” in the organism [31]. This implies that circadian regulation only functions optimally if feeding and thereby activity of the liver pacemaker is timed during the light period and thereby activity of the SCN pacemaker.

### The interdependence of sleep and circadian function

Sleep is mainly regulated by neurons producing the neuropeptides hypocretin-1 and -2 (HO), necessary for stabilizing wakefulness, and neurons producing melanin concentrating hormone (MCH), which are intermingled with the HO-neurons and are active during sleep (reviewed by Walker and Borniger [6]). These neurons, present in the lateral hypothalamus, are mutually exclusive, meaning that the activity of the HO-neurons inhibits the MCH-neurons and vice versa. The importance of HO-neurons for maintaining wakefulness is exemplified in people suffering from narcolepsy, an autoimmune disease that specifically targets the HO-neurons [32]. HO-neurons stimulate rapid corticosteroid production and release via activation of the HPA-axis [33], thereby mediating arousal, and are also an activator of the sympathetic nervous system [34]. Combined, these mechanisms are leading to daytime physiology and synchronization of peripheral clocks (Fig. 1). The functions of HO- and MCH-neurons, as well as other neuronal subsets involved in sleep regulation, are affected by systemic inflammation [6], which, as discussed later in this article, is commonly associated with cancer. As such, cancer may directly affect the systems controlling sleep, but sleep can also be indirectly affected through other mechanisms such as anxiety, pain, and nausea (Fig. 1).

The mounting evidence linking sleep and circadian disruption to both cancer incidence, progression, treatment outcome, and quality of life of the patients demands close consideration of sleep and circadian aspects in oncological practice. While many different tools, instruments, and tests have been used to measure or characterize sleep and circadian rhythms in cancer patients, consensus is lacking regarding the strengths and weaknesses of these techniques, as well as whether a minimal number or type of parameters should be included in the clinical assessment in order to achieve high-quality diagnosis of the sleep and circadian disruption. In this review, we therefore discuss commonly used techniques including biomarker analyses, subjective reporting systems/questionnaires, physiological reporting systems, and imaging-based systems to assess sleep and circadian disruption. We highlight the apparent strengths and weaknesses of these systems and discuss potential avenues for combining tests to improve accuracy of the outputs. We further examine the pathophysiological consequences of impaired sleep and circadian rhythms in the patients and delineate the known, underlying mechanisms, as well as efforts to maintain or restore circadian rhythmicity in patients to avoid circadian disruption-related complications or quality of life impairment. Aspects connecting the circadian clock to tumor metabolism will be discussed in detail as these pathways are intimately intertwined both at the cellular and endocrine levels.

## Recent advances in how sleep and circadian disruption can be diagnosed in cancer patients

Sleep disorders include a broad range of sleep-related phenotypes commonly found in cancer patients and which can be associated to the disease and/or treatment. These include restless legs syndrome and other sleep movement disorders, hypersomnolence (i.e., sleepiness during the day even after a full night’s sleep), sleeping-disordered breathing, including sleep apnea, and insomnia [2]. While each of these conditions has specific criteria that can be employed for accurate diagnosis (reviewed by Belachandran et al. [2]), they are commonly grouped under the umbrella “sleep disruption.” These conditions should, however, not be mistaken for other closely related quality of life indicators such as cancer-related fatigue, which is a different but often comorbid condition, that has a distinct etiology and mechanism, as recently reviewed by Saligan et al. [35].

A variety of techniques have been used to identify and measure the extent of sleep disruptions and/or circadian function in humans. These include imaging techniques, devices that register physiological parameters such as cardiovascular rhythms or movement, biomarkers of sleep or circadian disruption, including genetic, serum, urinary and salivary biomarkers, and subjective sleep assessment instruments and questionnaires.

### Imaging techniques

Various types of neuroimaging approaches have been deployed to study the neurobiology of sleep including magnetic resonance imaging (MRI), blood oxygen level dependent (BOLD) functional (f)MRI, single-photon emission computed tomography (SPECT), positron emission tomography (PET), and magnetization transfer imaging (MTI) [36, 37]. MRI and PET are mainly used to investigate the structure of the brain and thereby provide information on anatomical and physiological parameters such as reduced gray matter volume, which is seen in patients with sleep apnea and cortical arousal associated with sleep disruption [38, 39]. BOLD fMRI and SPECT, on the other hand, provide functional information related to brain activity and can therefore be used to study the activation of various sleep centers during the different sleep phases [39]. Clinical studies have employed structural MRI-based imaging protocols to analyze structural or functional changes in the brain of cancer patients associated with cognitive impairment (including sleep disruption) coupled to disease or therapy (reviewed by Amidi and Wu [40]). These studies consistently report reduced gray matter volume or density in cancer patients, which are further reduced after systemic medical chemotherapy [40], recapitulating the results from neuroimaging studies on patients with sleep disorders. Studies using fMRI, SPECT, PET, or MTI to investigate functional changes in the brain coupled to sleep disruption in cancer patients are, however, lacking. Such studies would, however, be important to demonstrate whether sleep disruption may influence the functional connectivity between the various brain regions involved in sleep differently in cancer patients compared to non-cancer patients and may elucidate the impact of chemotherapy on such connectivity networks. This would likely provide new and highly interesting insights into the neurobiology of sleep disruption in this patient group.

While neuroimaging studies have led to a wealth of information related to the neurobiology of sleep and have diagnostic potential, these techniques are highly resource-intensive and unpractical and might even be unethical if used for routine clinical diagnosis of sleep disruption in large groups of patients such as cancer patients.

### Physiological parameters

Numerous physiological parameters are associated with sleep and circadian rhythmicity including neurological, cardiovascular, metabolic, and neuromuscular function [1]. Most of these are measured in a procedure known as polysomnography, currently considered the gold standard in sleep medicine. Polysomnography is performed by recording electrical signals from electrodes mounted at key positions on the scalp (including at the muscles controlling eye movements), body, and limbs, combined with measuring nasal air-flow and peripheral blood oxygen saturation. This provides information on all physiological parameters of sleep and circadian rhythmicity including detailed analysis of brain activity waves associated with different sleep stages, eye movement associated with rapid eye movement (REM) sleep, cardiovascular rhythms, respiration, skeletal muscle contractions, and blood oxygen saturation, which are all reduced during sleep, but also affected by sleep disruptions [41]. Early studies found that “deep sleep” referred to as slow-wave sleep was greatly reduced or absent in cancer patients [42], and more recently, slow-wave sleep was found to be unchanged by medical treatment [43]. These findings were cooperated in a study demonstrating that both REM and slow-wave sleep were reduced in cancer patients, although REM was reduced to a lesser degree compared to slow-wave sleep. The study further concluded that cancer patients in general have difficulties both staying awake during the day and maintaining sleep during the night [44]. In a study including 12 multiple myeloma patients, Enderlin et al. found that sleep efficiency (e.g., the ability to stay asleep) was poor in these patients, as evidenced by frequent limb movements and low arterial oxygen saturation during the night. This could be improved slightly following two cycles of chemotherapy [45], suggesting that medical therapy would improve sleep quality in this patient group. Rosen and Brand also found that sleep disturbances were common among pediatric cancer patients, where the type of sleep disturbance was found to be cancer-specific (e.g., daytime sleepiness for CNS cancers but insomnia for leukemias) and developed mainly after diagnosis and treatment [46]. Combined, these and other studies demonstrate the power of polysomnography evaluation of cancer patients to gain detailed information related to sleep quality and how sleep and circadian physiology are disrupted in multiple and often complex ways in cancer patients, suggesting that a more in-depth understanding of the sleep issues among individual patients and personalization of sleep-correcting measures are required. As polysomnography is performed in specialized sleep labs and requires experienced personnel for achieving both correct recordings and interpretation of the recorded data, these techniques are, however, not routinely performed in cancer patients.

To simplify the procedure, smaller systems able to record the position and acceleration (i.e., movement) of a limb such as the arm, and that fit like a watch on the wrist of an individual, have been developed and implemented widely in sleep studies on cancer patients. Because of their facile implementation into clinical practice, such wrist-worn accelerometers have been widely used to produce a surrogate measurement of sleep (reviewed by Peddle-McIntyre CJ et al. [47]). Several different brands including Actigraph, SenseWear, ActivPAL, RT3-Triaxial, and more recently Fitbit have been used, but the majority of studies done to date are using the Actigraph (and commonly the GT3X) device [47]. The main output from these accelerometers is the displacement of the device, i.e., physical activity/arm movement. The device also records its own vertical or horizontal position and as such indicates when the individual is lying down. This is used to record when the person is in bed. From this information, various sleep/circadian-related parameters can be derived including the duration of periods without movement while in bed (the sleep latency), the total time of no movement (i.e., the total sleep time), the proportion of total sleep time compared to the total time in bed (the sleep efficiency), the time being awake after sleep onset, and the dichotomy index I < O indicating the percentage of all activity periods recorded while in bed that are lower than the median activity level during the active period (values closer to 100% representing rest and thereby stronger circadian function) [48]. Among these parameters, Palesh et a.l [48] found that the dichotomy index, the most widely used function to measure circadian function and sleep quality with these devices, is the only parameter that is significantly correlated (inversely) to the subjective experience of having sleep problems by the patient. Similarly, while the combination of subjective and objective scoring of poor sleep was the most accurate predictor of poor quality of life parameters such as poor social function, fatigue, and appetite loss, subjective sleep scoring by the patients was a better predictor compared to objective scoring using Actigraphy, if either measure was used alone [48]. This suggests that the value of objective measurements of circadian function using accelerometers provides a valuable complement to subjective instruments but is not sufficiently accurate to be used as an isolated technique.

### Biomarkers

Disturbed circadian rhythms influence immune functions which in turn affects duration and intensity of sleep [49]. Inflammation is an important circadian-related aspect of immune function, which can be diagnosed if the patient has elevated levels of circulating inflammatory cytokines such as CRP—a biomarker that is commonly associated with sleep disorders [50] and toxicity from chemotherapy [51]. Choshen-Hillel et al. [52] detected a low-grade inflammation seen as elevated CRP in individuals with chronic sleep disruption and deprivation. In healthy individuals with lower sleep and REM latency, elevated levels of inflammatory mediators such as IL-1 and IL-6 were detected [53]. Interestingly, in this study, the results were independent of the age, gender, or ethnicity of the patients, suggesting that inflammatory cytokines might be robust markers of sleep disruption even in a heterogeneous patient group. Indeed, inflammatory cytokines such as IL1β, IL4, IL6, IL10, TNFα, and TGFβ are known to be deregulated in cancer patients and affect the course of the disease, but also exert direct effects on the sleep neurocircuitry [54]. Interestingly, the pro-inflammatory Th1-associated cytokines IL1β, IL6, and TNFα are associated with a reduced proportion of REM (superficial) sleep and correspondingly increased NREM or slow-wave (deep) sleep [6]. The circulating levels of these cytokines are increased in cancer patients but also in healthy, sleep-deprived individuals. On the other hand, anti-inflammatory Th2-associated cytokines including IL4, IL10, and TGFβ induce wakefulness and inhibit both superficial and deep sleep [55]. As the circulating levels of pro-inflammatory and anti-inflammatory cytokines exhibit diurnal variation with a shift towards pro-inflammatory cytokines during the day and anti-inflammatory cytokines during the night, it is intriguing to speculate that augmented production of both classes of cytokines in cancer patients may contribute to their inability to both stay awake during the day and asleep during the night (Fig. 1). Further studies are required to investigate this hypothesis and to gain a deeper understanding of the mechanisms by which these cytokines affect the neurocircuitry of sleep.

In addition to inflammatory cytokines, circulating levels of the circadian hormones melatonin and cortisol are central outputs of sleep but also regulators of the sleep–wake cycle themselves. Lower levels of melatonin were associated with lower sleep quality in healthy female nurses working night shifts compared with age-matched female nurses working the day shifts [56]. Furthermore, Mazzoccoli et al. [57] found that elderly patients with advanced stage of lung cancer exhibited decreased melatonin levels and melatonin/cortisol ratios in the serum compared to healthy, age-matched controls. Also in a study on oral cancer patients, reduced salivary melatonin levels were found in the patient population compared to healthy controls, and while the patients also had poorer sleep compared to the controls, there was no association between sleep quality and salivary melatonin, which, however, could be due to the low number of patients (34 cancer patients and 33 controls) included in this study [58]. A review of the literature on breast cancer patients furthermore revealed that circadian rhythm in melatonin is maintained in the patients, but night-time peaks are lower compared to healthy controls and further disrupted after surgery and/or medical treatment [59]. As a key output of restorative night-time sleep, melatonin plays pleiotropic roles in cancer. Melatonin effectively inhibits epithelial to mesenchymal transition (EMT) and therefore tumor dissemination and metastasis, via down-regulation of pro-inflammatory IL-1β/NF-κB/MMP2/MMP9 pathways, extracellular matrix remodelling [60, 61], and mesenchymal gene expression, in both lung and breast cancer cells [62, 63]. As such, circadian hormones play important roles in mediating both directions of the crosstalk between sleep and cancer (Fig. 1).

Studies to elucidate genetic biomarkers of sleep disruption-associated negative health impact (including cancer) have mainly focused on the understanding the role of the core circadian clock genes. James et al. identified altered Per1 and Per2 expression in peripheral blood mononuclear cells (PBMCs) in shift workers after shifted sleep/wake schedules [64], suggesting that these genes may provide a link between sleep disruption and the pro-inflammatory state that is often regulated by these cells. Viola et al. found that polymorphisms in clock genes such as in *Per3* and more specifically the number tandem-repeats/length of the transcript were associated with sleep loss and affect individual sleeping patterns within the general population [65]. This did not, however, affect the circadian rhythms of circulating melatonin or cortisol. Bioinformatics approaches to identify transcriptomic signatures associated with sleep disruption has suggested that circadian phase, which is shifted or absent in patients with disrupted sleep, can be diagnosed by RNA sequencing of blood collected at a single time point, thereby avoiding the need for repeated sampling across the circadian cycle of the patient [66, 67]. While these studies were performed in healthy patients with undisrupted circadian cycles, should their findings hold true also for cancer patients with sleep disruptions, this would greatly facilitate the use of genomic/transcriptomic analysis as a diagnostic principle in the future.

Furthermore, mutations in or deregulated expression of circadian clock genes are frequently found in tumor cells and associated to carcinogenesis and malignancy [22]. As such, Clock protein and mRNA levels are upregulated and associated with estrogen receptor (ER)α signaling in breast cancer [68]. In gastric cancer, single nucleotide polymorphisms in Clock or Bmal1 affected their expression and were associated with poor clinical outcome [69]. Furthermore, patients with thyroid cancer expressed higher levels of Clock, Bmal1, and Per2 and reduced levels of Cry2 compared to age-matched cancer-free controls, which correlated to the extent of sleep disruption measured by the Pittsburgh sleep quality index in these patients [70].

### Subjective instruments and questionnaires

To measure sleep disorder in cancer patients and its consequences on quality of life, patient-reported experiences are often measured using validated scales (Table 1). A variety of such instruments exist, which have been developed for specific patient groups and conditions and are therefore targeted to specific aspects of sleep disruption found in these populations. None of the dedicated sleep instruments has been developed to assess sleep disruption in cancer patients, across indications, but instruments developed for other patient groups have been widely used and validated also for use in the oncology setting.

**Table 1:** Self-reported and fast questionnaires often used to measure sleep disturbances

Scale	Contents	Applicable in
Pittsburgh sleep quality index (PSQi)	19 items within seven domains. Measures retrospective sleep quality and disturbances over a 1-month period	Primarily used in psychiatry and insomnia disorders
The European organization for research and treatment of cancer (EORTC) quality of life questionnaire (EORTC-QLQ-C30)	30 items in five functional measures: physical, role, emotional, social, and cognitive	Used in cancer research and measures health related quality of life (HRQOL)
Medical outcomes study (MOS) sleep scale	12 items which measures sleep in 6 domains: initiation, quantity, maintenance, respiratory problems, perceived adequacy, somnolence	Patients with chronic disorders such as primary care, congestive heart failure and cancer populations
Functional outcomes of sleep questionnaire (FOSQ)	30-item questionnaire and a short version of 10 items. Contains 6 domains about general productivity, activity level, vigilance, social outcomes, and intimacy and sexual relationships	Often used in research and in clinical practice to measure the impact of daytime sleepiness on activities of daily living
Morningness-eveningness questionnaire (MEQ)	19 items related to sleep preferences and at what time the patients would like to carry out different activities	Used broadly in both healthy and patient populations
Munich chronotype questionnaire	7 questions to examine wake and sleep schedules (on both work and free days) to provide information about the individual’s circadian clock	Used broadly in both healthy and patient populations
The sleep timing questionnaire (STQ)	It is a diary for 7 days to assess the sleep pattern and sleepiness during the week	Useful in palliative care research and to assess the effect of therapies
Epworth sleepiness scale (ESS)	8 questions to assess daytime fatigue and the likelihood that a person will suffer from a sleep disorder	Used in outpatient care, in clinical practice, and in research
Stanford sleepiness scale even called for "Alertness Test"	1 item asking how sleepy the patient feels	Used for frequent resampling or in situations where simple and fast answers are needed
Insomnia severity index (ISI)	7 items to assess the type, severity, and impact of insomnia	Used to monitor response to sleep therapy in adults including cancer patients with insomnia

#### Pittsburgh sleep quality index (PSQi)

The PSQi is a commonly used instrument used for research on sleep disruption in the oncology setting. The questionnaire consists of 19 items within seven domains: sleep quality, sleep latency, sleep duration, habitual sleep efficiency, sleep disturbances, use of sleeping medications, and daytime dysfunction. It was developed for use in psychiatric practice and research [71] but has also been widely applied to the general population as a tool to gauge the prevalence of insomnia [72].

#### European organization for research and treatment of cancer (EORTC) quality of life questionnaire (EORTC-QLQ30)

The EORTC-QLQ30 questionnaire is used broadly to assess various aspects of quality of life in cancer patients and has been translated to more than 100 languages. It includes two questions related to sleepiness and sleep disruption [73]. This instrument has been widely used to assess cancer-related fatigue, but given the paucity of questions directly related to sleep, it should not be used in isolation to characterize the details and extent of sleep disruption.

#### Medical outcomes study (MOS) sleep scale

The MOS sleep scale was developed to assess sleep problems in a broad population of patients suffering from chronic illness, including cancer [74]. The instrument combines 12 questions assessing various aspects of sleep and daytime tiredness, answered on a 6-level Likert scale, and includes two questions providing a subjective assessment of the average time required to fall asleep and the hours slept during the night.

#### Functional outcomes of sleep questionnaire (FOSQ)

The FOSQ is used to assess disease-related quality of life which determines functional status and measures the extent and impact of excessive sleepiness on various activities of everyday living and the extent to which these activities are improved by effective treatment [75, 76]. This questionnaire exists in a long (30 items) and a short (10 items) form and evaluates the severity of each item on a 4–6-level Likert scale.

#### Morningness-eveningness questionnaire (MEQ)

The MEQ includes 19 items related to when the respondent prefers to sleep, wake-up, and do certain tasks or how the respondent would feel doing certain tasks at specified times during the morning, day, evening, or night, on a 4-level Likert scale. The output is an assessment of the “morningness” or “eveningness” phenotype of the respondent and thereby indicates the respondents “chronotype” [77, 78]. One landmark application of the questionnaire in cancer patients was within the Nurse’s Health Study, where people self-reporting as neither morning nor evening chronotypes had a 27% increased breast cancer incidence compared to those identifying as morning chronotypes [79].

#### Munich chronotype questionnaire (MCTQ)

Also the MCTQ is a self-test comprised of 17 questions evaluated on a 6-level Likert scale, used to evaluate individual sleep–wake preferences on work and work-free days [80, 81]. The questionnaire has been used in cancer patients to assess the correlation between self-identified chronotype and risk of developing cancer-related fatigue [82].

#### The sleep timing questionnaire (STQ)

The STQ is used by patients to report when they go to bed and get up in the morning and as such provides information on the bedtime habits of the individual. The questionnaire does not include a Likert scale, and the patients are instead required to write the actual time into the questionnaire [83].

#### Epworth sleepiness scale (ESS)

The ESS asks the respondent to, on a 4-level Likert scale, judge how likely he or she is to doze off in 8 different situations. This is used to grade the level of daytime sleepiness and has been used in cancer patients to assess sleep disruption [84].

#### Stanford sleepiness scale (SSS)

The SSS is the fastest and simplest instrument used to assess sleepiness as it contains only one item—a self-rating 7-point scale of how sleepy the responded feels in the exact moment he or she is answering. This simplified approach has been used in palliative care research [85] and to assess the effect of therapies to improve sleep quality in cancer patients [86].

#### Insomnia severity index (ISI)

The ISI is one of the most popular instruments used to assess the type, severity, and impact of insomnia as well as to monitor the response to sleep-correcting therapy in adults [87]. The ISI has been specifically validated for assessment of insomnia in cancer patients and to what extent this can be corrected through treatment [88]. It contains 7 items answered on a 5-level Likert scale.

### The need for subjectivity

To determine the presence and severity of sleep disorders objectively and correlate this to the patients’ experience of their sleep habits, a Norwegian research group compared self-reported experience to more objective measurement with polysomnography (PSG) or actigraphy. The result indicated a clear lack of agreement between the methods. The role of the objective techniques is still unclear, and most studies rely on patient-reported outcomes.

The reasons why cancer patients develop sleep and therefore circadian disruption are complex and may depend on many different factors such as pain from the tumor, anxiety, lack of physical activity, lack of social contacts, and side effects of the anticancer treatment. Furthermore, biological changes in the body due to both the tumor and treatment, such as inflammatory signaling or disruption of the blood–brain barrier in the brain regions controlling sleep, could play a role. Patients suffering from sleep disturbances already prior to diagnosis are, however, more likely to have further deteriorated sleep quality after diagnosis and treatment [89]. This suggests that non-cancer-related factors also play a role and that sleep disruption is not a transient condition only associated with diagnosis-associated stress and anxiety or acute toxicities from the treatment.

## Update on the known consequences of sleep/circadian disruption

The impact of sleep and/or circadian disruption on cancer patients is two-fold: First and foremost, the quality of life of the patient is often dramatically impaired due to insomnia and excessive daytime sleepiness. This is in turn leading to a lack of health-promoting actions such as exercise, social interactions, a healthy diet, and affirmative cultural or intellectual expressions. Secondly, sleep and circadian function are also coupled to robust treatment outcome and extended survival in cancer patients. Impaired circadian rest-activity rhythms have a general prevalence of 30–55% of cancer patients, where weaker rhythms (i.e., near-equal activity during the day and night) in the patients are correlated to more advanced and aggressive cancer, need for chemotherapy, poor treatment outcome, increased treatment-related toxicity, low quality of life, and poor survival [90–92]. A clear example of this was found in patients with advanced colorectal cancer where robust circadian activity rhythms, measured by Actigraphy, indicate better survival and improved quality of life [15]. Also in head and neck cancer, patients suffering from poor sleep were found to have reduced survival due to insufficient treatment response [93]. In breast cancer, sleep disruption is more prevalent in patients where the tumor cells have lost the short allele of the Per3 gene, but retained the long allele, leading to reduced overall expression of Per3 [94]. Furthermore, these Per3-low cancers are more aggressive, and the patients have a shorter relapse-free survival compared to patients with wild-type Per3. Across indications, reduced expression of Bmal1 by the tumor cells or specific mutations in Clock has been associated with progression or poor survival [22]. Similarly, the output from Bmal1/Clock signaling, i.e., Per1-3, is also commonly reduced in cancer cells and associated with more advanced disease and poor prognosis. On the other hand, Cry proteins are often found to be elevated in tumor cells due to de-repression upon reduction in Per levels [34]. Genetic markers have also been used by Lu et al. [95] to identify responding patient populations with rectal cancer. In this study, Clock, Cry2, and Per2 expression levels in the tumor cells were elevated in patients obtaining a complete response to radiochemotherapy. This study did not, however, investigate progression or sleep disturbances in the patients. Moreover, a general dampening of the circadian oscillations in gene expression was found in breast cancer tissue compared to healthy tissue from the same patient [96], further strengthening the functional impact of the disruption of the circadian clock genes in cancer cells.

### Chronomodulation of therapy

In addition to ensuring high-quality sleep, treatment should be timed according to the expression/function of the process or molecular pathway they target. Such chronomodulation both improves therapeutic treatment outcome and minimizes toxicity (reviewed by Levi et al. [17]). The appropriate timing of administration for a number of cancer treatments has now been elucidated but remains unknown for the majority of the most commonly used drugs [17]. As the circadian function (the chronotype) of individual patients likely differ [12], a novel concept within personalized medicine includes an individual assessment of the patients’ chronotype. Combining such information with the known chronopharmacology of the indicated drugs, this would help the oncologist to understand the most efficient time for administration of the treatment for the specific patient [97]. The importance of such an approach was recently highlighted in a preclinical study investigating the timing of an anticancer therapy in wild-type and clock-mutant mice [98]. Anticancer drugs exhibit time-of-day dependent efficacy in cells with a functional clock and should be given at the time-of-day when they are most effective. In clock-deficient cells, the efficacy of the drugs was, however, impaired, suggesting that also for patients, understanding the circadian function of the tumor cells could help to predict medical treatment outcome.

### Normalizing sleep and circadian function in cancer patients

As disrupted and poor quality sleep have detrimental effect on quality of life, efforts have been made to alleviate the problem, and various types of interventions have been investigated (Table 2). In clinical practice, pharmacological products to promote sleep (hypnotics) are commonly used [99]. As these medicines have unwanted side effects and create an artificial sleep, there have been trials exploring other, non-pharmacological methods. There is good evidence for sleep and circadian rhythm restoring effects of mind–body exercises in particular for breast cancer patients [100]. Also physical exercise, although often used mainly to prevent or ameliorate cancer-related fatigue, has been shown to have good efficacy also for managing insomnia in these patients (reviewed by Armstrong et al. [99]). Similarly, a large review exploring the possible effect of yoga on several quality of life parameters included a total of more than 2100 patients in 24 studies [101]. They found moderate-quality evidence for a short-term positive effect on sleep disturbances. Aromatherapy as a stress-relieving aid during the sleep phase may also lead to a significant improvement in sleep quality [102]. A more physiological approach based on treatment with bright light for 30 min per day was found to significantly improve sleep and circadian function in breast cancer patients [103], suggesting that an over-exposure to light during the day can help normalize the circadian function of the patient.

**Table 2:** Interventions to improve sleep and circadian function in cancer patients

Intervention	Patient group	Ref
Hypnotics	General	99
Melatonin	Breast cancer, lung cancer	110, 111
Physical activity	General	99
Mind–body exercises	Breast cancer	100
Yoga	General	101
Aroma therapy	Multiple	102
Light therapy	Breast cancer	103
Cognitive behavioral therapy for insomnia (CBT-I)	General	104, 105, 106

While sedatives/hypnotics are commonly used for treatment of sleep disruptions [99], the 2016 national comprehensive cancer network guidelines recommend against this practice, at least if used as a monotherapy [104]. Sedatives/hypnotics can, however, be beneficial when combined with cognitive and behavioral therapy for insomnia (CBT-I). CBT-I is, however, recommended and more frequently used as a single intervention to treat insomnia in cancer patients [99, 104]. The use of CBT-I for improving sleep in cancer patients has a strong support in the literature, as recently evidenced in several meta-analyses [105, 106].

A key molecular consequence of sleep disruption is reduced melatonin production during the night, and as such, it is not surprising that exogenous administration of melatonin has been used to treat the effects of sleep and circadian disruption [107, 108]. In adults, and especially in the elderly population, melatonin has minimal if any toxicity [109] and may both improve sleep in cancer patients (hypnotic effects) as well as resynchronize circadian clocks (chronobiotic effects) [110] and as such increase the efficacy of (chronomodulated) chemotherapy [111].

In summary, the problem of sleep and circadian disruption in cancer care is multifaceted. Both the understanding of whether and to what extend a patient suffers from sleep disruption, as well as possible actions to help the patients overcome such issues in evidence-based clinical practice, are highly warranted.

## What are the important mechanisms underlying the pathological effects of sleep/circadian disruption in cancer patients—including metabolic disruption

As discussed in the preceding sections, sleep disruptions and circadian dysregulation have profound effects on cancer patients and their treatments. The underlying mechanisms and how these can be targeted to improve treatment outcomes and quality of life in cancer patients with circadian disruptions remain, however, poorly understood. Cortisol is a circadian hormone directly produced through stimulation of the HPA-axis by HO-neurons during the day and known to be a major regulator of circadian rhythmicity (Fig. 1). Tumors may disrupt the brain clock, leading to non-circadian HPA-axis activation and disrupted cortisol release [112]. A potential mechanism by which cancers may affect the brain clocks is by secreting cytokines that lead to neuroinflammation, blood–brain barrier breakdown, and thereby circadian rhythm disruption and sleep disruption [113]. Initially, cortisol levels may be elevated after one or a few nights of poor sleep, leading to immune-suppression and increased risk of infection. After longer time with circadian disruption, however, adrenal exhaustion is leading to reduced cortisol levels. Cortisol treatment is a common addition to other cytostatics or sometimes even used as the main anticancer treatment, for example, in patients with lymphomas. As no studies have investigated the effects of cortisol in poor versus good sleepers, it is however unclear whether the effects of cortisol treatment may or may not be coupled to its role on circadian resynchronization.

### Circadian regulation of tumor metabolism

The circadian clock is intricately involved in regulation of cellular metabolism and regulates processes leading to the preferential use of glucose and glycolytic pathways for energy production during the day but mainly oxidative phosphorylation during the night (recently reviewed by Kanouchi and Sassone-Corsi [22]). This further implies that oxidative stress is high during the day but ameliorated during the night. Should, however, the cellular clock be deregulated, such as seen in many types of cancer, this generally leads to expansion of day-like metabolism through the night phase and therefore excessive glycolytic flux and oxidative stress in the cells [51]. For example, Npas2, a core clock gene involved in the positive limb of the clock and active mainly during the day, directly upregulates genes critical for glycolysis such as HIF1α, Glut1, Hk2, Aldoa, and Eno2 and downregulates Pgc1α in hepatocellular carcinoma [114], which accelerated the growth and metastasis of these tumors in vivo (Fig. 2). The circadian disruption found in cancer cells is thereby critical for maintaining a perpetual state of aerobic glycolysis (Warburg effect), characterized by glutamine oxidation [115], lipogenesis, and nucleotide synthesis—thereby contributing to the metabolic hallmarks of cancer [116]. These pathways are also regulated by the circadian clock in non-malignant cells. For example, reduced expression of NAMPT, the rate-limiting enzyme in the NAD + salvage pathway, and consequently reduced NAD + levels and glycolysis were found in the livers of mice lacking Clock or Bmal1 [117, 118], further strengthening the central role of the circadian clock machinery in regulating cellular metabolism.

In addition to the cellular clock, endocrine circadian signaling is also involved in regulating tumor metabolism. Aberrant HO (hypocretin) signaling from the brain through the SNS in patients having poor quality sleep drives metabolic changes in the tumor cells including increased glycolysis [119]. This could be blocked, and healthy sleep could be restored by the hypocretin (HO)-receptor antagonist almorexant. As HO is critical for wakefulness, and elevated upon sleep deprivation, this explains how sleep disruption signals from the brain may induce metabolic changes similar to the Warburg effect in the tumor cells. In addition to the Warburg effect in well perfused and therefore oxygenated parts of the tumor, local hypoxia in poorly perfused areas is also an important contributor to glycolytic metabolic profiles of the cells. In this context, glycolysis-mediated acidification drives inhibition of the mammalian target of rapamycin complex 1 (mTORC1), which in turn suppresses the circadian clock in the cells [120]. As discussed previously, there is extensive crosstalk between hypoxia-inducible and circadian clock transcription factors within the cells [23, 121] providing a further mechanism for suppression of circadian function in hypoxic tumor cells. Genetic disruption of the clock in the tumor cells may, however, also contribute to the Warburg effect. Bmal1, Per2, and histone H3 at promoters of circadian genes are rhythmically deacetylated by NAD + -dependent Sirt1 [122–124], and this process is absent in Sirt1-knock out mice leading to low and stable (non-cycling) levels of the circadian clock factors. Also other metabolites play important roles in maintaining cellular circadian function. Nucleotide-diphosphate-N-acetylglucosamine (Nuc-GlcNAc) [125–127] inhibits ubiquitinylation of Bmal1/Clock, increasing their half-life and thereby inhibiting establishment of robust rhythms, S-adenosylmethionine (SAM) and flavin adenine dinucleotide (FAD) [128], instead increase the stability of clock factors such as Cry in the negative limb of the clockwork. As these factors are strongly and rapidly regulated by blood glucose levels, the metabolic state of an individual is central to the organization of circadian rhythms in the cells, including cancer cells. Recently, Verlande et al. [129] described that serum glucagon drives development of muscle and fat wasting syndrome (cachexia) by destabilization of the circadian clock factor Rev-Erbα, which acts as a negative regulator of gluconeogenesis, thereby increasing hepatic glucose production. As such, circadian disruption in cancer patients may not only lead to pathological metabolic changes in the tumor cells but also detrimental catabolic responses in healthy tissues of the patients. An interesting approach to reinstating circadian rhythmicity in both the tumor cells as well as the healthy tissues is to employ a “temporal feeding” regimen including intermittent fasting during the evening and night. Such an approach has shown efficacy in mouse cancer models, where tumor growth was impaired by restricted meal-timing [130, 131]. As breast cancer risk increases in women who are eating at night compared to only during the day [132], restricting meal times to only during the day may also be an attractive and simple method to manage circadian disruption in cancer patients in the future.

### Effects of the circadian clock on tumor angiogenesis

Tumor angiogenesis is critical for tumor growth and dissemination [133, 134]. Vascular endothelial growth factor (VEGF) is the most well-described pro-angiogenic factor involved in tumor angiogenesis [135], which in turn is induced by a variety of pathophysiological aspects of the tumor microenvironment including inflammation and hypoxia. We have recently found that Bmal1 and Per2 directly regulate transcription of the VEGF gene in an opposing manner, where Bmal1 induces and Per2 inhibits transcription via binding to E-boxes present in the VEGF promoter [24, 136, 137]. This leads to rhythmic production of VEGF preferentially during the night. It is intriguing to hypothesize that deregulated circadian clocks in tumor cells likely also leads to deregulated VEGF-production and in turn continuous rather than cyclic stimulation and growth of the tumor vasculature. This would lead to an absence of VEGF-free vascular maturation periods, which might be contributing to the pathologically immature nature of tumor blood vessels, and increase the risk metastatic dissemination. This hypothesis should be further investigated in the future as it may also impact on the timing of anti-angiogenic drugs given to cancer patients.

## Conclusions and future outlook

It is now undisputed that sleep disruption is a common and growing health issue in modern societies and that sleep disruption should be considered a carcinogenic factor. Sleep disruption leads to a non-circadian lifestyle in which sleep, activity, and food intake are taking place both during the day and during the night, or frequently shift between these two circadian periods. This in turn leads to a loss of circadian physiology which perhaps most clearly is observed as excessive daytime sleepiness coupled to insomnia at night. Such phenotypes are not only associated with an increased risk of cancer but also frequently seen in the cancer patient population itself, and patients that are more severely affected by sleep or circadian disruption have worse prognosis compared to those having robust sleep and without circadian dysfunction. As such, it is no longer a question of whether we should analyze the sleep physiology and circadian function of people as a part of regular health checks, screening programs or after a cancer diagnosis, but rather how we should do it. Future guidelines on health promotion and disease prevention should address this issue, but guidelines for specifically analyzing the extent of sleep and circadian disruption in cancer patients should also be implemented. We recommend that objective techniques such as Actigraphy or fitbit accelerometers are combined with subjective techniques such as validated questionnaires, rather than relying solely on objective techniques, to provide the most accurate assessment. If the available resources do not allow such a rigorous analysis, subjective instruments seem to provide more accurate data on sleep disruption in the patients compared to objective sleep-devices, and subjective instruments should therefore be prioritized in a single test-only context. As the majority of cancer patients may suffer from sleep disruption to a varied extent, accurate diagnosis and treatment of this comorbidity should not be ignored. This is particularly important as evidence has accumulated in recent years demonstrating that sufficient, restorative sleep may have significantly stronger therapeutic effects when combined with medical treatment than any medical treatment alone. Sleep medication should, however, not become the primary sleep-correcting intervention, but cognitive behavioral therapy, mind–body exercises, or physical exercise should be employed, as this seems to have much greater efficacy in cancer patients. Furthermore, understanding the patients’ chronotype and adjusting the administration of the medical treatments accordingly (i.e., chronomodulated therapy) should become an integrated practice in oncology clinics. To achieve this, preclinical research is required to identify biomarkers that may be employed to accurately assess a patients’ chronotype and to understand the mechanisms by which the circadian clock influences the efficacy of anticancer therapies.

We are beginning to understand the importance of circadian disruption in cancer and the underlying mechanisms. We, however, still have a long way to go in terms of delineating the complex interplay between factors regulating circadian physiology and implementing a sleep restoration and medical chronomodulation mindset in the clinics. Efforts in this regard, however, have already had significant impact on improving the treatment efficacy, survival, and quality of life of the patients and could be one of our greatest hopes for, with relatively simple means, achieving even higher-quality care for our patients in the future.

## References

[1] Allada R, Bass J (2021). Circadian mechanisms in medicine. New England Journal of Medicine.

[2] Balachandran DD, Miller MA, Faiz SA, Yennurajalingam S, Innominato PF (2021). Evaluation and management of sleep and circadian rhythm disturbance in cancer. Current Treatment Options in Oncology.

[3] Roenneberg T (2013). Chronobiology: The human sleep project. Nature.

[4] Bin YS, Postnova S, Cistulli PA (2019). What works for jetlag? A systematic review of non-pharmacological interventions. Sleep Medicine Reviews.

[5] Kripke DF (2015). Genetic variants associated with sleep disorders. Sleep Medicine.

[6] Walker, W.H., 2nd & Borniger, J.C. (2019) Molecular Mechanisms of Cancer-Induced Sleep Disruption. *Internation Journal Molecular Science*, *20*, 2780. 10.3390/ijms20112780 PMC6600154 31174326

[7] Wegrzyn LR (2017). Rotating night-shift work and the risk of breast cancer in the Nurses' Health Studies. American Journal of Epidemiology.

[8] group, I.M.V. (2019). Carcinogenicity of night shift work. Lancet Oncol.

[9] Walker, W.H., 2nd*, et al.* (2020) Light pollution and cancer. *Internation Journal Molecular Science*, *20*, 9360. 10.3390/ijms21249360 PMC7764771 33302582

[10] Hadadi E (2020). Chronic circadian disruption modulates breast cancer stemness and immune microenvironment to drive metastasis in mice. Nature Communications.

[11] Yennurajalingam S (2018). Factors associated with patient-reported subjective well-being among advanced lung or non-colonic gastrointestinal cancer patients. Palliative & Supportive Care.

[12] Palesh OG (2010). Prevalence, demographics, and psychological associations of sleep disruption in patients with cancer: University of Rochester Cancer Center-Community Clinical Oncology Program. Journal of Clinical Oncology.

[13] Phillips KM, Jim HS, Donovan KA, Pinder-Schenck MC, Jacobsen PB (2012). Characteristics and correlates of sleep disturbances in cancer patients. Supportive Care in Cancer.

[14] Otte JL (2009). Comparison of subjective and objective hot flash measures over time among breast cancer survivors initiating aromatase inhibitor therapy. Menopause.

[15] Innominato PF (2012). Prediction of overall survival through circadian rest-activity monitoring during chemotherapy for metastatic colorectal cancer. International Journal of Cancer.

[16] Ikoma N, Raghav K, Chang G (2017). An update on randomized clinical trials in metastatic colorectal carcinoma. Surgical Oncology Clinics of North America.

[17] Levi F, Okyar A, Dulong S, Innominato PF, Clairambault J (2010). Circadian timing in cancer treatments. Annual Review of Pharmacology and Toxicology.

[18] Oliva D (2017). Single nucleotide polymorphisms might influence chemotherapy induced nausea in women with breast cancer. Clin Transl Radiat Oncol.

[19] Oliva D, Sandgren A, Nilsson M, Lewin F (2014). Variations in self-reported nausea, vomiting, and well-being during the first 10 days postchemotherapy in women with breast cancer. Clinical Journal of Oncology Nursing.

[20] Dallmann R, Brown SA, Gachon F (2014). Chronopharmacology: New insights and therapeutic implications. Annual Review of Pharmacology and Toxicology.

[21] Ruben MD, Smith DF, FitzGerald GA, Hogenesch JB (2019). Dosing time matters. Science.

[22] Kinouchi K, Sassone-Corsi P (2020). Metabolic rivalry: Circadian homeostasis and tumorigenesis. Nature Reviews Cancer.

[23] Jensen LD (2015). The circadian clock and hypoxia in tumor cell de-differentiation and metastasis. Biochimica et Biophysica Acta.

[24] Jensen LD, Gyllenhaal C, Block K (2014). Circadian angiogenesis. Biomol Concepts.

[25] Hogenesch JB, Gu YZ, Jain S, Bradfield CA (1998). The basic-helix-loop-helix-PAS orphan MOP3 forms transcriptionally active complexes with circadian and hypoxia factors. Proc Natl Acad Sci U S A.

[26] Ghorbel MT, Coulson JM, Murphy D (2003). Cross-talk between hypoxic and circadian pathways: Cooperative roles for hypoxia-inducible factor 1alpha and CLOCK in transcriptional activation of the vasopressin gene. Molecular and Cellular Neuroscience.

[27] Bunger MK (2000). Mop3 is an essential component of the master circadian pacemaker in mammals. Cell.

[28] Dimova EY (2019). The circadian clock protein CRY1 Is a negative regulator of HIF-1alpha. iScience.

[29] Kobayashi M (2017). A circadian clock gene, PER2, activates HIF-1 as an effector molecule for recruitment of HIF-1alpha to promoter regions of its downstream genes. FEBS Journal.

[30] Reppert SM, Weaver DR (2001). Molecular analysis of mammalian circadian rhythms. Annual Review of Physiology.

[31] Dibner C, Schibler U, Albrecht U (2010). The mammalian circadian timing system: Organization and coordination of central and peripheral clocks. Annual Review of Physiology.

[32] Latorre D (2018). T cells in patients with narcolepsy target self-antigens of hypocretin neurons. Nature.

[33] Bonnavion P, Jackson AC, Carter ME, de Lecea L (2015). Antagonistic interplay between hypocretin and leptin in the lateral hypothalamus regulates stress responses. Nature Communications.

[34] Geerling JC, Mettenleiter TC, Loewy AD (2003). Orexin neurons project to diverse sympathetic outflow systems. Neuroscience.

[35] Saligan LN (2015). The biology of cancer-related fatigue: A review of the literature. Supportive Care in Cancer.

[36] Tahmasian, M.*, et al.* (2021) ENIGMA-Sleep: Challenges, opportunities, and the road map. *Journal Sleep Research, 30*, e13347. 10.1111/jsr.13347 PMC8803276 33913199

[37] Hardy SJ, Krull KR, Wefel JS, Janelsins M (2018). Cognitive changes in cancer survivors. American Society of Clinical Oncology Educational Book.

[38] Hermesdorf, M.*, et al.* (2021) Sleep characteristics, cognitive performance, and gray matter volume: findings from the BiDirect Study. *Sleep, 44,* zsaa209. 10.1093/sleep/zsaa209 33029624

[39] Campabadal A, Segura B, Junque C, Iranzo A (2021). Structural and functional magnetic resonance imaging in isolated REM sleep behavior disorder: A systematic review of studies using neuroimaging software. Sleep Med Rev.

[40] Amidi A, Wu LM (2019). Structural brain alterations following adult non-CNS cancers: A systematic review of the neuroimaging literature. Acta Oncologica.

[41] Rundo JV, Downey R (2019). Polysomnography. Handbook of Clinical Neurology.

[42] Silberfarb PM, Hauri PJ, Oxman TE, Lash S (1985). Insomnia in cancer patients. Social Science and Medicine.

[43] Roscoe JA (2011). Few changes observed in polysomnographic-assessed sleep before and after completion of chemotherapy. Journal of Psychosomatic Research.

[44] Parker KP (2008). Sleep/Wake patterns of individuals with advanced cancer measured by ambulatory polysomnography. Journal of Clinical Oncology.

[45] Enderlin CA (2013). Sleep measured by polysomnography in patients receiving high-dose chemotherapy for multiple myeloma prior to stem cell transplantation. Oncology Nursing Forum.

[46] Rosen G, Brand SR (2011). Sleep in children with cancer: Case review of 70 children evaluated in a comprehensive pediatric sleep center. Supportive Care in Cancer.

[47] Peddle-McIntyre CJ (2018). A review of accelerometer-based activity monitoring in cancer survivorship research. Medicine and Science in Sports and Exercise.

[48] Palesh O (2017). Relationship between subjective and actigraphy-measured sleep in 237 patients with metastatic colorectal cancer. Quality of Life Research.

[49] Besedovsky L, Lange T, Haack M (2019). The sleep-immune crosstalk in health and disease. Physiological Reviews.

[50] Razeghi E, Sahraian MA, Heidari R, Bagherzadeh M (2012). Association of inflammatory biomarkers with sleep disorders in hemodialysis patients. Acta Neurologica Belgica.

[51] Liu L (2012). Fatigue and sleep quality are associated with changes in inflammatory markers in breast cancer patients undergoing chemotherapy. Brain, Behavior, and Immunity.

[52] Choshen-Hillel S (2021). Acute and chronic sleep deprivation in residents: Cognition and stress biomarkers. Medical education.

[53] Mills PJ (2007). Inflammation and sleep in healthy individuals. Sleep.

[54] Opp MR (2005). Cytokines and sleep. Sleep Medicine Reviews.

[55] Obal F, Krueger JM (2003). Biochemical regulation of non-rapid-eye-movement sleep. Frontiers in Bioscience.

[56] Wu X (2021). Circadian rhythm disorders and corresponding functional brain abnormalities in young female nurses: A preliminary study. Frontiers in neurology.

[57] Mazzoccoli G, Carughi S, De Cata A, La Viola M, Vendemiale G (2005). Melatonin and cortisol serum levels in lung cancer patients at different stages of disease. Medical science monitor : international medical journal of experimental and clinical research.

[58] Salaric I (2021). Salivary melatonin in oral squamous cell carcinoma patients. Science and Reports.

[59] Ahabrach, H., El Mlili, N., Errami, M. & Cauli, O. (2020) Circadian rhythm and concentration of melatonin in breast cancer patients. *Endocrine Metabolism Immune Disorder Drug Targets, 10,* 1869-81. 10.2174/1871530320666201201110807 33261546

[60] Wang X (2018). Melatonin inhibits epithelialtomesenchymal transition in gastric cancer cells via attenuation of IL1beta/NFkappaB/MMP2/MMP9 signaling. International Journal of Molecular Medicine.

[61] Reiter, R.J.*, et al.* (2017) Melatonin, a full service anti-cancer agent: Inhibition of initiation, progression and metastasis. *Internation Journal Molecular Science*, *18*, 843. 10.3390/ijms18040843 PMC5412427 28420185

[62] Chao CC (2019). Melatonin suppresses lung cancer metastasis by inhibition of epithelial-mesenchymal transition through targeting to Twist. Clinical Science (London, England).

[63] Mao L (2016). Melatonin represses metastasis in Her2-postive human breast cancer cells by suppressing RSK2 expression. Molecular Cancer Research.

[64] James FO, Cermakian N, Boivin DB (2007). Circadian rhythms of melatonin, cortisol, and clock gene expression during simulated night shift work. Sleep.

[65] Viola AU (2007). PER3 polymorphism predicts sleep structure and waking performance. Current biology : CB.

[66] Laing, E.E.*, et al.* (2017) Blood transcriptome based biomarkers for human circadian phase. Blood transcriptome based biomarkers for human circadian phase. *Elife, 6*, e20214. 10.7554/eLife.20214 PMC5318160 28218891

[67] Braun R (2018). Universal method for robust detection of circadian state from gene expression. Proc Natl Acad Sci U S A.

[68] Xiao L (2014). Induction of the CLOCK gene by E2-ERα signaling promotes the proliferation of breast cancer cells. PloS one.

[69] Chen Y (2019). Functional polymorphisms in circadian positive feedback loop genes predict postsurgical prognosis of gastric cancer. Cancer medicine.

[70] Lou X (2021). Alterations of sleep quality and circadian rhythm genes expression in elderly thyroid nodule patients and risks associated with thyroid malignancy. Scientific reports.

[71] Buysse DJ, Reynolds CF, Monk TH, Berman SR, Kupfer DJ (1989). The Pittsburgh sleep quality index: A new instrument for psychiatric practice and research. Psychiatry Research.

[72] Backhaus J, Junghanns K, Broocks A, Riemann D, Hohagen F (2002). Test-retest reliability and validity of the Pittsburgh sleep quality index in primary insomnia. Journal of Psychosomatic Research.

[73] Kaasa S (1995). The EORTC core quality of life questionnaire (QLQ-C30): Validity and reliability when analysed with patients treated with palliative radiotherapy. European Journal of Cancer.

[74] Bower JE (2000). Fatigue in breast cancer survivors: Occurrence, correlates, and impact on quality of life. Journal of Clinical Oncology.

[75] Weaver TE (1997). An instrument to measure functional status outcomes for disorders of excessive sleepiness. Sleep.

[76] Ferrer M (1999). Measurement of the perceived impact of sleep problems: The Spanish version of the functional outcomes sleep questionnaire and the Epworth sleepiness scale. Medicina Clínica (Barcelona).

[77] Horne JA, Ostberg O (1976). A self-assessment questionnaire to determine morningness-eveningness in human circadian rhythms. International Journal of Chronobiology.

[78] Taillard J, Philip P, Chastang JF, Bioulac B (2004). Validation of Horne and Ostberg morningness-eveningness questionnaire in a middle-aged population of French workers. Journal of Biological Rhythms.

[79] Ramin C (2013). Chronotype and breast cancer risk in a cohort of US nurses. Chronobiology International.

[80] Roenneberg T, Wirz-Justice A, Merrow M (2003). Life between clocks: Daily temporal patterns of human chronotypes. Journal of Biological Rhythms.

[81] Juda M, Vetter C, Roenneberg T (2013). The Munich chronotype questionnaire for shift-workers (MCTQShift). Journal of Biological Rhythms.

[82] Starreveld DEJ (2021). Cancer-related fatigue in relation to chronotype and sleep quality in (non-)Hodgkin lymphoma survivors. Journal of Biological Rhythms.

[83] Monk TH (2003). Measuring sleep habits without using a diary: The sleep timing questionnaire. Sleep.

[84] Tag Eldin ES, Younis SG, Aziz L, Eldin AT, Erfan ST (2019). Evaluation of sleep pattern disorders in breast cancer patients receiving adjuvant treatment (chemotherapy and/or radiotherapy) using polysomnography. J BUON.

[85] Good P, Pinkerton R, Bowler S, Craig J, Hardy J (2018). Impact of opioid therapy on sleep and respiratory patterns in adults with advanced cancer receiving palliative care. Journal of Pain and Symptom Management.

[86] Kim, H., Lee, Y.W., Ju, H.J., Jang, B.J. & Kim, Y.I. (2019) An exploratory study on the effects of forest therapy on sleep quality in patients with gastrointestinal tract cancers. An Exploratory Study on the Effects of Forest Therapy on Sleep Quality in Patients with Gastrointestinal Tract Cancers. *International Journal Environmental Research Public Health, 16*, 24490. 10.3390/ijerph16142449 PMC6678486 31295818

[87] Bastien CH, Vallieres A, Morin CM (2001). Validation of the insomnia severity index as an outcome measure for insomnia research. Sleep Medicine.

[88] Savard MH, Savard J, Simard S, Ivers H (2005). Empirical validation of the insomnia severity index in cancer patients. Psycho-Oncology.

[89] Fleming, L.*, et al.* (2019) Insomnia in breast cancer: a prospective observational study.* Sleep, 42*, zsy245. 10.1093/sleep/zsy245 30521041

[90] Milanti, A., Chan, D.N.S., Li, C. & So, W.K.W. (2021) Actigraphy-measured rest-activity circadian rhythm disruption in patients with advanced cancer: A scoping review. *Support Care Cancer, 29, *7145-7169. 10.1007/s00520-021-06317-3 34142279

[91] Soucise A (2017). Sleep quality, duration, and breast cancer aggressiveness. Breast Cancer Research and Treatment.

[92] Wiggins EK (2020). Sleep quality and prostate cancer aggressiveness: Results from the REDUCE trial. Prostate.

[93] Cash E (2018). Depressive symptoms and actigraphy-measured circadian disruption predict head and neck cancer survival. Psycho-Oncology.

[94] Fores-Martos J (2021). Circadian PERformance in breast cancer: A germline and somatic genetic study of PER3(VNTR) polymorphisms and gene co-expression. NPJ Breast Cancer.

[95] Lu H (2015). Circadian gene expression predicts patient response to neoadjuvant chemoradiation therapy for rectal cancer. International Journal of Clinical and Experimental Pathology.

[96] Lesicka M (2018). Altered circadian genes expression in breast cancer tissue according to the clinical characteristics. PLoS One.

[97] Hesse, J.*, et al.* (2020) An optimal time for treatment-predicting circadian time by machine learning and mathematical modelling.* Cancers (Basel), 12*, 3103. 10.3390/cancers12113103 PMC7690897 33114254

[98] Lee, Y.*, et al.* (2021) Time-of-day specificity of anticancer drugs may be mediated by circadian regulation of the cell cycle. *Science of Advanced, **7*, abd2645. 10.1126/sciadv.abd2645 PMC7880601 33579708

[99] Armstrong TS (2017). Sleep-wake disturbance in patients with brain tumors. Neuro-Oncology.

[100] Kreutz C, Schmidt ME, Steindorf K (2019). Effects of physical and mind-body exercise on sleep problems during and after breast cancer treatment: A systematic review and meta-analysis. Breast Cancer Research and Treatment.

[101] Cramer H (2017). Yoga for improving health-related quality of life, mental health and cancer-related symptoms in women diagnosed with breast cancer. Cochrane Database Syst Rev.

[102] Ozkaraman A, Dugum O, Ozen Yilmaz H, Usta Yesilbalkan O (2018). Aromatherapy: The Effect of Lavender on Anxiety and Sleep Quality in Patients Treated With Chemotherapy. Clin J Oncol Nurs.

[103] Wu HS, Davis JE, Chen L (2021). Bright light shows promise in improving sleep, depression, and quality of life in women with breast cancer during chemotherapy: Findings of a pilot study. Chronobiology International.

[104] Denlinger CS (2016). NCCN guidelines insights: Survivorship, Version 1.2016. J Natl Compr Canc Netw.

[105] Johnson JA (2016). A systematic review and meta-analysis of randomized controlled trials of cognitive behavior therapy for insomnia (CBT-I) in cancer survivors. Sleep Medicine Reviews.

[106] Garland SN (2014). Sleeping well with cancer: A systematic review of cognitive behavioral therapy for insomnia in cancer patients. Neuropsychiatric Disease and Treatment.

[107] Pfeffer M, Korf HW, Wicht H (2018). Synchronizing effects of melatonin on diurnal and circadian rhythms. General and Comparative Endocrinology.

[108] Auld F, Maschauer EL, Morrison I, Skene DJ, Riha RL (2017). Evidence for the efficacy of melatonin in the treatment of primary adult sleep disorders. Sleep Medicine Reviews.

[109] Andersen LP, Gogenur I, Rosenberg J, Reiter RJ (2016). The safety of melatonin in humans. Clinical Drug Investigation.

[110] Innominato PF (2016). The effect of melatonin on sleep and quality of life in patients with advanced breast cancer. Supportive Care in Cancer.

[111] Lissoni P, Chilelli M, Villa S, Cerizza L, Tancini G (2003). Five years survival in metastatic non-small cell lung cancer patients treated with chemotherapy alone or chemotherapy and melatonin: A randomized trial. Journal of Pineal Research.

[112] Aiello, I., Mul Fedele, M.L., Roman, F.R., Golombek, D.A. & Paladino, N. (2021) Circadian disruption induced by tumor development in a murine model of melanoma. *Chronobiology International, 39*, 1–14. 10.1080/07420528.2021.1964519 34482768

[113] Mampay M, Flint MS, Sheridan GK (2021). Tumour brain: Pretreatment cognitive and affective disorders caused by peripheral cancers. British Journal of Pharmacology.

[114] Yuan P (2020). Circadian clock gene NPAS2 promotes reprogramming of glucose metabolism in hepatocellular carcinoma cells. Cancer Letters.

[115] Tohyama S (2016). Glutamine oxidation Is Indispensable for survival of human pluripotent stem cells. Cell Metabolism.

[116] Vander Heiden MG, DeBerardinis RJ (2017). Understanding the intersections between metabolism and cancer biology. Cell.

[117] Nakahata Y, Sahar S, Astarita G, Kaluzova M, Sassone-Corsi P (2009). Circadian control of the NAD+ salvage pathway by CLOCK-SIRT1. Science.

[118] Ramsey KM (2009). Circadian clock feedback cycle through NAMPT-mediated NAD+ biosynthesis. Science.

[119] Borniger JC (2018). A role for hypocretin/orexin in metabolic and sleep abnormalities in a mouse model of non-metastatic breast cancer. Cell Metab.

[120] Walton ZE (2018). Acid suspends the Circadian clock in hypoxia through inhibition of mTOR. Cell.

[121] Tabebi M, Soderkvist P, Jensen LD (2018). Hypoxia signaling and circadian disruption in and by pheochromocytoma. Front Endocrinol (Lausanne).

[122] Nakahata Y (2008). The NAD+-dependent deacetylase SIRT1 modulates CLOCK-mediated chromatin remodeling and circadian control. Cell.

[123] Asher G (2008). SIRT1 regulates circadian clock gene expression through PER2 deacetylation. Cell.

[124] Hirayama J (2007). CLOCK-mediated acetylation of BMAL1 controls circadian function. Nature.

[125] Fustin JM (2013). RNA-methylation-dependent RNA processing controls the speed of the circadian clock. Cell.

[126] Kaasik K (2013). Glucose sensor O-GlcNAcylation coordinates with phosphorylation to regulate circadian clock. Cell Metabolism.

[127] Li MD (2013). O-GlcNAc signaling entrains the circadian clock by inhibiting BMAL1/CLOCK ubiquitination. Cell Metabolism.

[128] Hirano A, Braas D, Fu YH, Ptacek LJ (2017). FAD regulates CRYPTOCHROME protein stability and circadian clock in mice. Cell Reports.

[129] Verlande, A.*, et al.* (2021) Glucagon regulates the stability of REV-ERBalpha to modulate hepatic glucose production in a model of lung cancer-associated cachexia. *Science of Advanced, **7*, eabf388. 10.1126/sciadv.abf3885 PMC8232919 34172439

[130] Filipski E (2005). Effects of light and food schedules on liver and tumor molecular clocks in mice. Journal of the National Cancer Institute.

[131] Wu MW, Li XM, Xian LJ, Levi F (2004). Effects of meal timing on tumor progression in mice. Life Sciences.

[132] Li M (2017). Nighttime eating and breast cancer among Chinese women in Hong Kong. Breast Cancer Research.

[133] Cao Y (2005). Opinion: Emerging mechanisms of tumour lymphangiogenesis and lymphatic metastasis. Nature Reviews Cancer.

[134] Folkman J (1971). Tumor angiogenesis: Therapeutic implications. New England Journal of Medicine.

[135] Ferrara N (2009). Vascular endothelial growth factor. Arteriosclerosis, Thrombosis, and Vascular Biology.

[136] Jensen LD (2012). Opposing effects of circadian clock genes bmal1 and period2 in regulation of VEGF-dependent angiogenesis in developing zebrafish. Cell Reports.

[137] Koyanagi S (2003). A molecular mechanism regulating circadian expression of vascular endothelial growth factor in tumor cells. Cancer Research.

